# Three-Dimensional Computed Tomography Reconstruction for Revision Parathyroidectomy

**DOI:** 10.7759/cureus.1715

**Published:** 2017-09-26

**Authors:** Navdeep R Sayal, Ryan J Brisson, Kenneth Richey, Christine Lepoudre

**Affiliations:** 1 Department of Otolaryngology, Beaumont Health; 2 Oakland University William Beaumont School of Medicine; 3 William Beaumont Health System Imaging Services, Beaumont Health

**Keywords:** hyperparathyroidism, surgery, endocrine surgery, imaging

## Abstract

A 57-year-old man presented with persistent hyperparathyroidism following primary parathyroidectomy. A four-dimensional computed tomography scan with three-dimensional reconstruction showed two parathyroid glands (one right and one left) and anatomic variation from previous surgery. Revision surgery was performed revealing the parathyroid glands as expected from the preoperative three-dimensional reconstruction. After surgery, the patient recovered well, and preoperative symptoms resolved. The use of three-dimensional computed tomography reconstruction provided accurate localization of the parathyroid glands and surrounding anatomic structures. This resulted in decreased preoperative planning cost, operative time, and estimated blood loss typical for patients who have multiple preoperative imaging studies.

## Introduction

Hyperparathyroidism is a common cause of hypercalcemia that may cause renal failure, hypertension, fatigue, and mental status changes. Hyperparathyroidism commonly presents with concurrent pathology such as parathyroid adenomas (85% patients), hyperplasia (10% patients), multiple adenomas (4% patients), and carcinoma (1% patients) [1]. Surgery may provide definitive cure for hyperparathyroidism in >90% patients [2]. Surgery for primary hyperparathyroidism is a minimally invasive procedure that provides improved clinical and aesthetic results compared to bilateral neck exploration. Minimally invasive parathyroidectomy has a high success rate (99.5%) and low risk of morbidity (<1%) [3].

In 5 to 10% of patients, revision surgery may be required to treat recurrent or persistent hyperparathyroidism. Although revision parathyroid surgery is often successful, there may be a substantially increased risk of morbidity due to previous scarring and anatomic variation caused by the previous operation [3]. Surgical re-exploration has been shown to be associated with complications in 27% patients; only 89% patients are cured and 17% patients have permanent morbidity. This is typically because of difficulty associated with scarring and anatomic variation from previous surgery [3,4]. Therefore, preoperative planning is important to localize the parathyroid glands. It is important for the surgeon to be aware of surrounding structures such as the thyroid gland, larynx, trachea, recurrent laryngeal nerves, esophagus, and great vessels to minimize potential morbidity. Preoperative planning and localization imaging studies typically include high-resolution ultrasonography (sensitivity, 21%), magnetic resonance imaging (MRI) scanning, computerized tomography (CT) scanning, technetium (99mTc) sestamibi imaging (sensitivity, 54%), and four-dimensional (4D) CT scanning (sensitivity, 88%) [5]. In combination, these methods may help localize and guide the surgical approach. While these imaging modalities help localize parathyroid abnormalities, cases still exist where the parathyroid gland is difficult to find intraoperatively. Therefore, we utilized a three-dimensional (3D) reconstruction of a 4D CT scan to better allow us to visualize the parathyroid gland compared to the surrounding structures. To date, the authors have not found any previously reported cases using 3D reconstruction to aid in pre-operative planning for parathyroid surgery.

We treated a patient who had persistent hyperparathyroidism by using a 3D reconstruction of a 4D CT scan for preoperative planning for revision parathyroid surgery.

## Case presentation

A 57-year-old man who had primary hyperparathyroidism presented to the Ear, Nose, and Throat clinic because of fatigue and agitation. A 4D CT scan showed a 2.6 × 1.3 cm ovoid mass at the sternomanubrial junction with contrast enhancement on both arterial and portal venous phases in the area where a sestamibi parathyroid study showed focal activity. A right inferior substernal parathyroid adenoma was excised. The excised parathyroid was markedly enlarged and had adenomatous characteristics including hypercellularity and gross enlargement. Pathology examination of the surgical specimens showed a hypercellular area of parathyroid tissue that was surrounded by a fibrous capsule, distinct and separate from adjacent normocellular parathyroid tissue, characteristic of parathyroid adenomas. The surgery was believed to be successful.

**Figure 1:**
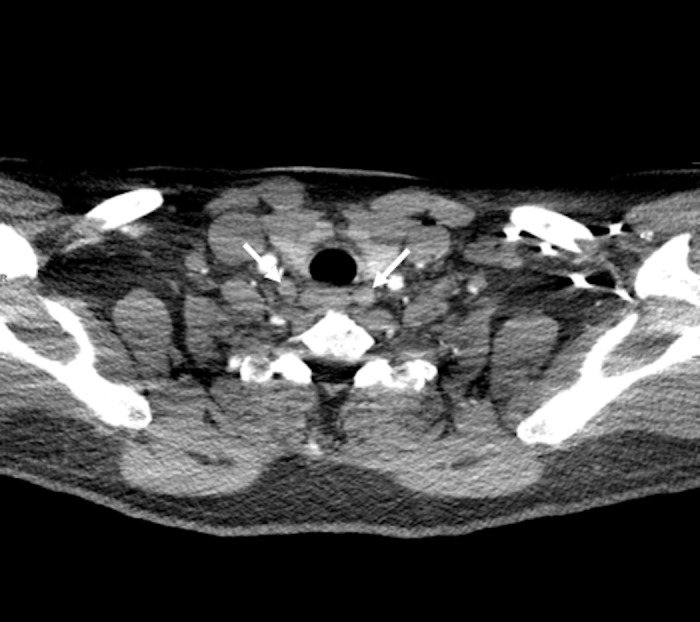
Axial Computed Tomography (CT) Scan of Parathyroid. A 57-year-old man had primary hyperparathyroidism. Preoperative parathyroid revision axial 4D-CT shows a 10 mm ovoid structure on the right and an 11 mm structure on the left side (white arrows).

**Figure 2:**
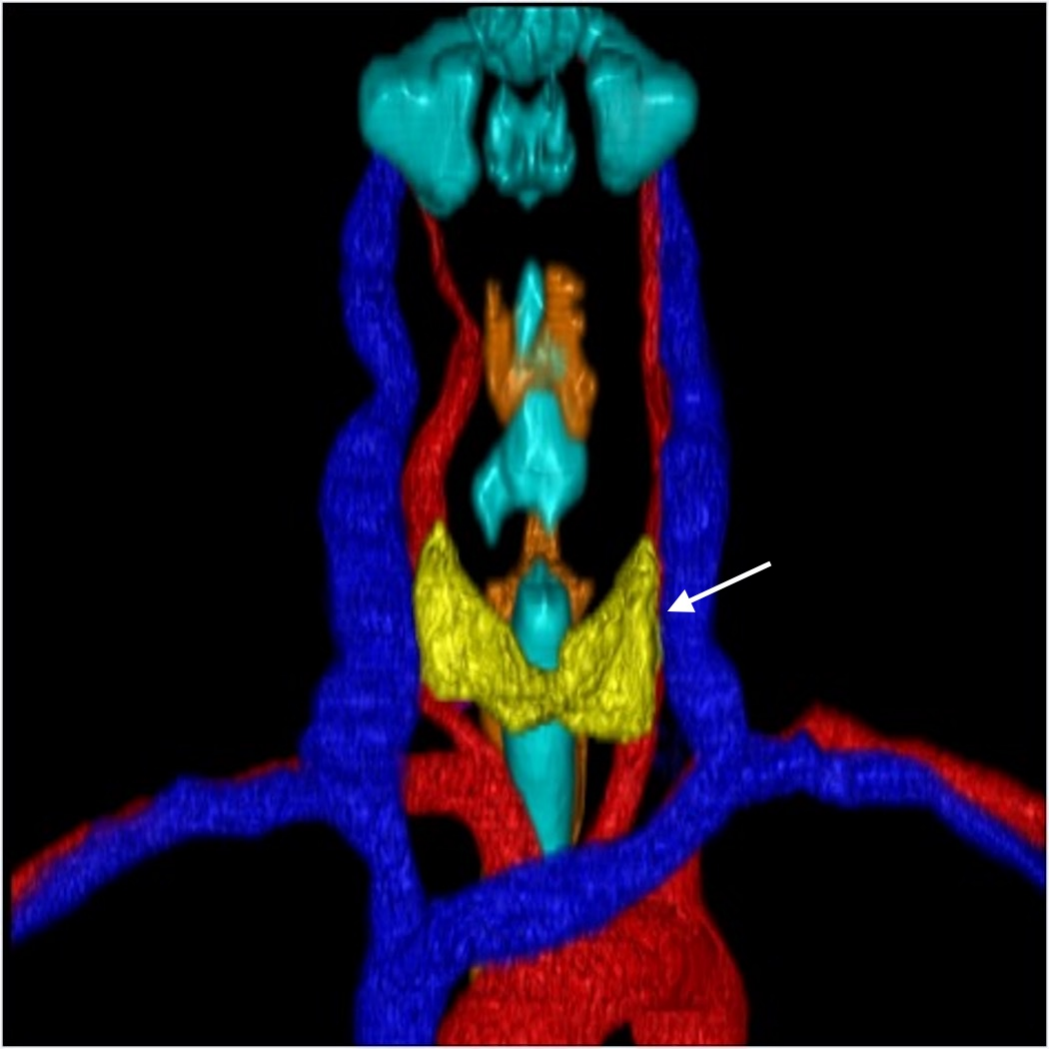
3D Reconstruction Anterior/Posterior View. Anteroposterior view of the thyroid gland (white arrow) and surrounding structures.

**Figure 3:**
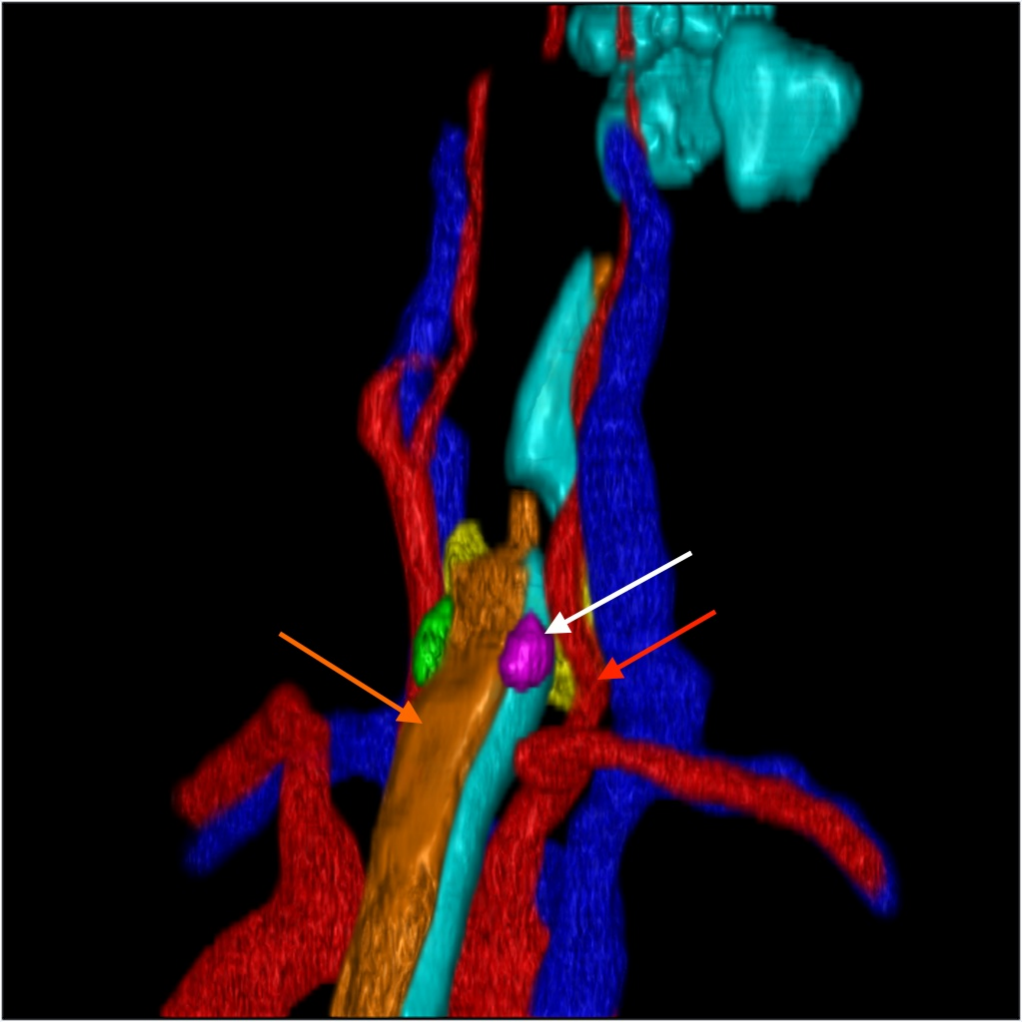
3D Reconstruction Right Oblique View. Right oblique view showing the right parathyroid gland (white arrow) in relation to the great vessels and esophagus. Notice the parathyroid just adjacent to the esophagus (orange arrow) and posteromedial to the carotid (red arrow).

**Figure 4:**
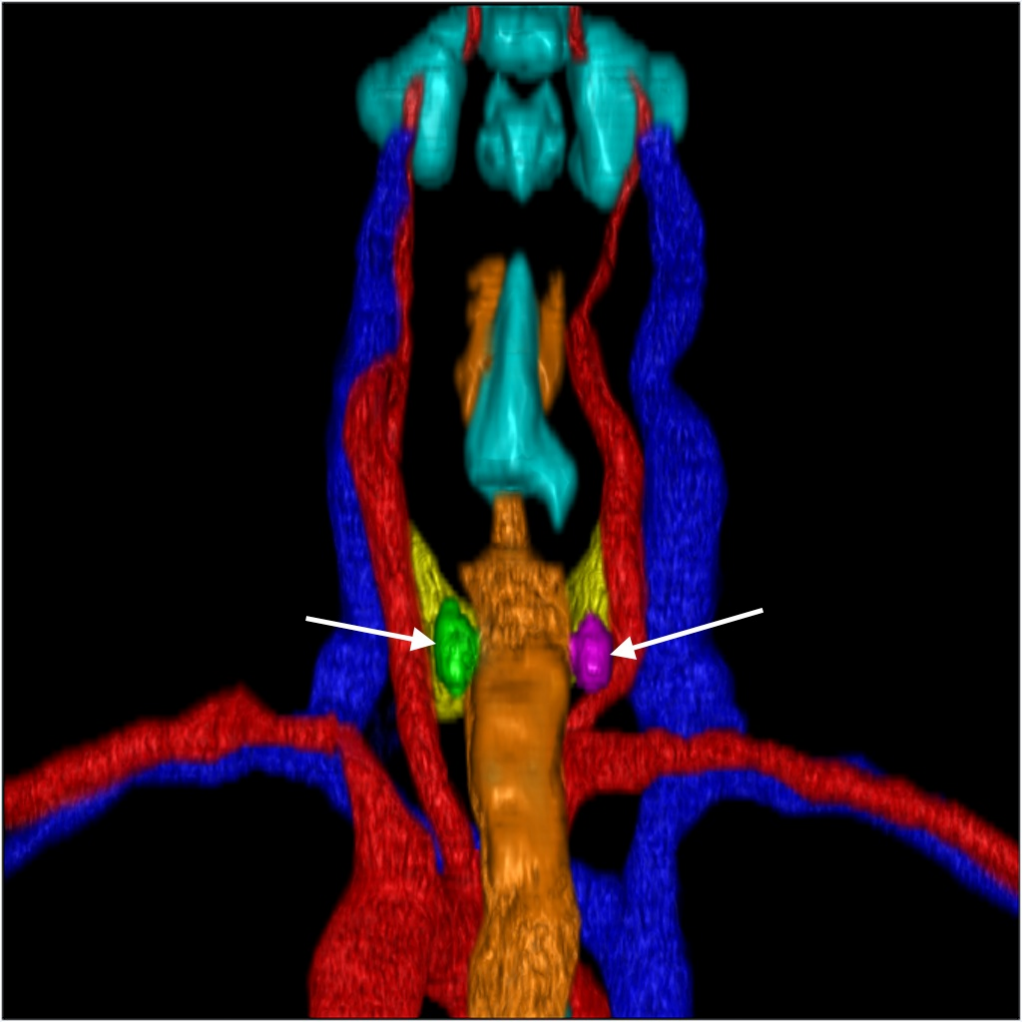
3D Reconstruction Posterior View. Posterior view showing vertical height differences between the left (green) and right (purple) parathyroid glands (also indicated by the white arrows).

The patient returned three months later because of extreme fatigue. The parathyroid hormone (PTH) level was found to be elevated to 304. The diagnosis of persistent hyperparathyroid disease was made and reoperation was recommended because of probable parathyroid gland hyperplasia. A 4D CT scan of the neck showed two parathyroid glands (one right and one left) (Figure 1). Three-dimensional reconstruction (Figures 2, 3, 4) sharply demonstrated the two parathyroid glands in relation to the other major structures. This approximated well the anatomic features of the glands, trachea, great vessels etc., which are a precise representation of the human structures. For example, the left parathyroid gland was more posteroinferior than the right parathyroid gland and the left parathyroid gland lay just lateral to tracheoesophageal groove. The 3D post-processing was performed by 3D imaging laboratory staff on the 4D CT scan for parathyroid gland surgical planning (Vital Images VES version 6.5.5, Minnetonka, Minnesota). Anatomic segmentation was obtained by 3D software automation and hand contouring of specific anatomy to localize the parathyroid glands with respect to surrounding anatomic structures including arterial vessels, venous vessels, trachea, esophagus, and thyroid gland. Skin surface rendering was included to provide location references in the x, y, and z planes and 3D box scale provided with the 3D imaging software. Each anatomic structure was given a unique color and transparency to provide rapid identification within the 3D model. Image batches were created with localization references and sent to the radiographic picture archiving and communication system for review and use on screen in the operating room.

At revision surgery, the parathyroid glands were localized as expected from the preoperative 3D imaging studies. The two parathyroid glands were tan, oval shaped, and similar to each other in size. Biopsies showed that the right and left parathyroid glands were hypercellular. After histologic confirmation of parathyroid tissue, intraoperative blood PTH level showed 50% reduction- 76. The total operative time was 48 minutes, and estimated blood loss was 10 milliliters. Pathology demonstrated hypercellular parathyroid tissue composed of chief cells that gradually transitioned to areas of normocellular parathyroid tissue and intermingled hypercellular areas composed of oncocytes. The patient had no postoperative complications and his preoperative symptoms resolved.

## Discussion

Although 4D CT scanning is helpful for preoperative lateralization and localization of the parathyroid glands and surrounding structures, it provides accurate localization in only 80% and lateralization in 89% revision cases [5]. The 3D reconstruction used in the present case provided an alternative method of location of the parathyroid glands and important anatomic structures in the surgical field. The present patient had successful intraoperative localization of parathyroid adenomas with the aid of 3D reconstruction from a 4D CT scan. The 3D reconstruction enabled improved spatial detail about the location of the parathyroid glands and served to simplify surrounding complex structures. The relationship between the parathyroid and thyroid glands was important to improve efficiency of the surgery. For example, the reconstruction image showed the left parathyroid was located slightly more superior to the right one, which we remembered while dissecting the right side. The 3D imaging enabled preoperative visualization and decision-making that typically are performed during the surgery which, in our opinion, decreased the length of the operation and blood loss. Operating room charges vary from different hospitals from $22 to $143 per minute [6]. The 3D imaging reconstruction from our institution costs approximately $100, therefore, even if it saves a few minutes in the operating room, this technology deserves consideration.

Limitations of the 3D technology in planning parathyroid revision surgery include potential limitations of accurate identification of the parathyroid glands on CT imaging. In the present case, CT imaging did not identify a fourth parathyroid gland, therefore, a 3D image could not be created. Although it is unlikely that this patient had less than four parathyroid glands (less than 3% individuals have a decreased number of parathyroid glands) we opened the carotid sheath, explored the mediastinum, and inspected the tracheoesophageal groove [7]. Nevertheless, 3D imaging had the advantages of improved preoperative imaging that contributed to the focused operative approach. Further research should be performed to assess and compare the operative time, blood loss during surgery, and cost to other modalities.

## Conclusions

In summary, the present case confirmed that 3D CT reconstruction may be a useful approach to preoperative planning for revision parathyroid surgery. 3D CT reconstruction provided excellent spatial localization of the parathyroid glands and surrounding anatomic structures to enable successful and efficient performance of potentially complex revision parathyroid surgery. The 3D CT reconstruction has the potential to decrease the cost of revision surgery, operative time, and blood loss.
